# Proceedings of American Academy of Dental Surgery

**Published:** 1876-03

**Authors:** 


### ARTICLE III.

### *American Academy of Dental Surgery.*

Regular Meeting, held November 16th, 1875.

The American Academy of Dental Surgery held its regular meeting on the evening of November 16th, Dr. Geo. H. Perine, President, in the chair. After the usual preliminary business had been attended to, Prof. Frank H. Hamilton, M. D., of New York, made an address upon "The subject of Dentistry." He said:

Gentlemen, when, in response to Dr. Perine's polite note, I accepted his invitation to meet you this evening, and to make some remarks, I supposed the meeting to be social and informal; and I was a good deal surprised to see it announced that I would "address" the Academy. Excuse me gentlemen, but the few remarks that I shall make, will be better characterized as a familiar "talk"—certainly they will not rise to the dignity of an "address."

As to the importance of dentistry—the profession you have chosen—there can be no difference of opinion. In a sanitary and æsthetic point of view, it holds a high rank. Good teeth are essential to health, and equally essential to the beauty and harmony of the human face; and perhaps the best evidence that we can have that the world in general, and Americans in particular, hold the same opinion, may be found in the fact, that you are so well patronized and so liberally paid. I do not think you are overpaid, for your duties are delicate and laborious; but then every one is not paid all he fairly earns.

I have not myself employed dentists often; but, as to that matter, I have seldom employed a doctor, although I have been educated to have a good deal of respect for doctors. The fact is gentlemen, I, in common with other surgeons, claim to be in some sense a dentist or dental surgeon, and in my "treatise on surgery," I have devoted a chapter to this subject. We are very closely related by *blood*, and we must naturally respect each other.

What I think we may discuss profitable is, *the qualifications which ought to be required of those who practice dentistry;* and that will depend entirely upon what dentistry or dental surgery, as you prefer to call it, and very properly, means. Most other specialties define themselves, and tell us with some precision what region or anatomical part they intend to occupy; but dentistry as a science, is new, and its limits do not seem to be yet accurately defined.

When I began to practice medicine and surgery, I had the whole field to myself. Like Robinson Crusoe, I owned the whole island. There were then no specialists worth speaking of. There were a few spine doctors, and cancer doctors and purely mechanical dentists, and that was about all. But since then, one after another, men with carpet bags in their hands and rifles on their shoulders, have come in, driven their stakes, and set up their claims. Without asking a question or saying "by your leave," they have taken possession of the eye, the ear, the jaws both upper and lower—the throat, the lungs, the heart, the kidneys, the bladder, the uterus, the rectum, the nerves, the joints of crooked backs, crooked legs, and crooked feet, until what was once only known as the physician and surgeon's territory, has been pretty much all taken up by squatters; and the original proprietors, who hunted and dug where-ever they chose, over the whole ground, but drove no stakes, find themselves dispossessed. No one has yet driven a stake into the nose, or the spleen, or the large and small intestines. The liver, also, is for the present unoccupied; but this is probably because it was thoroughly worked some years ago, and it was found not to pay, so it was abandoned. Possibly also, there may be something in a name, and men may not like to be called "billious" doctors, or "splenetic" doctors.

I will not discuss how much good or harm comes of this sub-division of labor. Doubtless some good and doubtless some harm. The specialist sees farther and more distinctly in a line straight before his eyes, or through his own telescope, than anyone else; but he is in danger of not seeing anything outside of the narrow cylinder through which he is looking. He sees, and perhaps very much magnified, the pustule in the ear, or the granulation upon the ostincæ, but he fails often to see the general dyscrasy, the great constitutional fault and perturbation, upon which these insignificant affairs depend. Notwithstanding all the good they have done in the world, this is one of their admitted dangers and defects; but specialists exist, (it is well for surgery that they do exist,) and this is the fact with which we have to deal.

Now as to the qualifications which ought to be demanded of dentists, that will depend upon what they undertake to do, or as I have said before, upon how you define dental surgery.

If the dentist practices merely the extraction of teeth—the filling of teeth, and the making and setting of teeth, why then he needs very little more than a mechanical training; but if he proposes to treat all the diseases and accidents to which the buccal cavity is liable, including the teeth and diseases and accidents of the jaws, then he needs as a foundation, a thorough knowledge of medicine and of surgery, including of course, the collateral sciences, anatomy, physiology pathology, &c. In other words, dental surgery is then but an outgrowth from general surgery and medicine—a mere branch, receiving its supplies from the common trunk—a craft if you please, but which by special care and attention, is made to bear more perfect fruit than the parent stock.

This is not requiring too much, since it is impossible to separate the diseases of a part of the body from the whole. It is no more than is required of the oculist, and aurist and of every other medical or surgical specialist. They are all required before they can be recognized as "regulars," to possess themselves of that amount of knowledge which is, or ought to be implied by the degree of Doctor in Medicine. This is the standard to which dental colleges must attain in their course of instructions, to entitle their graduates to be recognized by their sister professions, unless they intend them to practice exclusively, or almost exclusively mechanical dentistry.

While we welcome heartily all well qualified specialists, we are exceedingly jealous of our rights as against those who attempt to practice any branch of surgery without the proper qualifications. We regard them as intruders, and as dangerous pretenders.

If each of us, (for these remarks apply as much to myself as to you,) if all of us limit ourselves in our efforts to treat disease, and to practice surgery, to what we thoroughly understand, we shall do our duty, and do the most good in the world.

I do not understand that you entertain any different opinions. On the contrary, dentists have always in their conversations with me, deprecated the introduction of imperfectly qualified men. They have always held that those who profess to practice surgery in connection with mechanical dentistry, should do it only under the right conferred by the degree of Doctor in Medicine, or the degree of a dental college whose standard was equivalent to that of a regular medical college. Nor will it do to attempt to evade this just requisition, by accepting of an honorary degree, conferred without an examination, or any pretense of a proper course of studies.

These I understand, are your opinions; but the difficulty is in keeping out improper men, or in preventing those admitted solely as mechanical dentists, from assuming other and wholly incompatible functions.

I appreciate your difficulty, and do not intend to make any criticism upon your conduct as a profession. Indeed it would be very unbecoming in me to do so, since I am well aware that there are a great many incompetent men in my own profession; and they continue to come in. Any one who has stood at the door as long as I have, authorized to give tickets of admission, knows how difficult it is, when the crowd and the rush are so great, to keep out improper applicants. No doubt if we had a sufficient number of stout men at the various entrances, and the gates were always kept partly closed, so that only one man could enter at a time and we could have a good look at him as he was passing in, we might do better; but experience proves that it is not easy to find a sufficient number of stout men, able and willing to do this duty.

Gentlemen—let me say before I sit down, that as a profession, you have made within the last twenty-five years, most wonderful progress; and especially is this true of American dental surgeons. The world concedes this, and I shall not be accused of any attempt to stretch the wings of the American eagle by saying so. It seems entirely safe, therefore, to entrust the future of this science and art in your hands. Whether I am to call you children or brethren, or cousins, or whatever may be our relationship, I am proud of the connection and shall feel honored by your friendship.

Dr. J. R. Gober said that Dr. Hamilton's ideas agreed with his exactly as to what the dental profession ought to do. If they were dental surgeons, they belonged to a branch of the medical profession. The door into the medical profession was now open, and they had only to walk in, but, in order to walk in, they must qualify themselves to go in. He thought it was impossible to separate the two professions. There are so many diseases that only a dental surgeon could properly treat, that they could not limit their work to singly extracting and filling teeth and working artificial sets.

Dr. J. J. Ketchum, New York, said he had been very much interested in the paper of Prof. Hamilton. The question of dental qualifications had two sides, as well as every other question. The physician was at sea when he reached the mouth, and dealt with the teeth. He was then unable through any advantages which he could get from his text books, to differentiate in his diagnosis between affections that might be entirely different in themselves and might require different treatment. There was the other side of the question, too, and that was that the dental surgeon was called upon to differentiate none the less than the physician or surgeon; and in order to do that, he had not only to know the worth, but the whole system at large. A man could not possibly deal with a pathological condition unless he first had anatomy and physiology, and then pathology, before him. He must grasp the whole field before he could take in a part. The man who went into the profession of dentistry, and looked at these matters exclusively from the mechanical point of view, was not fit to deal with these pathological conditions; because there were some operations that the dentist had to do that required the nicest tact—over genius, to carry out. Dentists should begin at the bottom, and have their anatomy and physiology, and upon that broad basis of scientific information they should build up their pathology. Their profession was a high one; it certainly ranked as a specialty in medicine.

Dr. John Allen said the dentists were indebted to the medical profession for the progress they had made. What was their condition forty years ago? They had no dental literature, no dental associations, no dental colleges, nothing upon which they could base their claims upon the medical profession. Now all this was changed; the medical profession had yielded up to them that branch of their profession connected with dentistry, showing that they had entire confidence in the ability of the dentists to deal with it. The dental profession was based upon the medical profession. They depended upon the medical profession, and they ought to acknowledge that dependence. Where would they be without pathology, physiology and anatomy? The medical profession had done for the dentists far more than the dentists had done for them, and therefore dentists ought to be modest in pressing their claims upon them for consideration.

Dr. Geo. H. Perine said he had never felt more proud of his profession than he did at the present time, when he looked back over twenty-five years and thought of the "toil and trouble" with which they had built themselves up to their present position,—recognized as they now are as specialists in medicine.

A paper was then read on "Association," by Dr. G. A. Mills, Hartford, Ct., in which the Doctor urged the desirability in many operations, of a union of tin with gold.

Dr. A. P. Merrill, in the discussion that followed the paper, said he would like to add his testimony in favor of tin foil. He had used it in many instances, and always in combination with gold. He once used it in combination with gold from the force of circumstances. In 1863, when practising in South America, a long distance from any dental depot, he happened to get nearly out of gold, but had plenty of tin foil. A young lady, of an aristocratic family, was to be married, was to go some distance into the interior after the ceremony, and he was requested to put her teeth in a good condition. He said he did not have the proper material to fill her teeth with, but he was told to do the best he could. He accordingly filled the cavities with tin nearly to the surface, and filled the balance with gold.

About a year ago, a dentist, a South American friend of his, visited him, and, among other things, spoke of the case, and said the lady had never felt any ill effects from the filling. Since then he had performed a few similar operations, and had no reason to doubt that in many cases tin and gold could be used together with great advantage.

Dr. F. H. Burrows said his reminiscences of the practice of dentistry went back a great many years, and his earliest remembrances were of tin being used with great advantage. He could trace through the whole course of his practice, tin fillings which had been put in the earlier days of dentistry. Once in a while he would come across a tin filling covered with gold, which somebody had excavated, and said, "What a cheat!" But perhaps dentists then did not have the same aim in using tin that they now have.

There had been wonderful strides made in the profession. When they commenced, in 1830, they were hewers of wood and drawers of water. If a step was made in advance, those who knew of it would say to each other, "Hush! keep it quiet! don't let others know it, by any manner of means!" They used, for instance, split pins for clamps, and ordinary chalk instead of Plaster of Paris. The younger members had made great improvements. In the early days dentists had nobody to teach them, and had to depend upon themselves. He had his fingers croched many a time in carving old sea horse, and he had carved a tooth from the handle of a tooth brush.

With reference to tin combined with gold, he thought that Dr. Mills had struck the right vein. But tin was not the only metal that had been so combined with gold. One man who had been a dentist for over forty years, thought he had made a valuable discovery in regard to exposed nerves, when he put pure lead over the nerve, and filled up with gold. Now instead of oxychloride of zinc, for the same purpose, Dr. Mills was using tin. He had used tin himself in the case of an old physician, and the patient finally died with the tooth in his mouth. [Laughter.] He believed in anything that would prevent the sense of shock, which was very great in the application of gold fillings, as they were used at the present day. He knew tbere was not a great deal of respect for tin current; but there was nothing like having plenty of American tin, if one had it in the right spot. [Laughter.]

Dr. A. Johnson said that while he had no doubt that tin and gold could be sometimes used together very well, yet he failed to see any great advantage in the union. Tin would not work as readily as would the gold alone. They had other substances which were better non-conductors than tin to put over the sensitive part of the tooth, and which could be more easily manipulated, and better adapted to the tooth. He did not mean to disparage tin, but he preferred to use something else. For temporary teeth, he preferred to use gutta-percha or Hill's stopping. As to amalgam, there were cases where it could be used. When the tooth was very far gone, he then preferred to use amalgam, Hill's stopping or oxy-chloride. But he never said that they were as good as gold. He had an old friend whose teeth had been destroyed, without doubt, by amalgam. He had seen gold fillings that had stood in the mouth longer than any amalgam to his knowledge. He had lately seen a right central incisor that had been filled on its upper surface with gold, forty-one years before, and the filling was as good as it was on the day it was put in.

Dr. Geo. H. Perine said that while they all knew the value of association, he, for one, was not prepared to recognize any decided advantage to be gained by the association of tin with gold. At the same time, he comprehended the value of tin as a stopping; and he would say nothing against either tin or amalgam unless they were poor. Under certain conditions, with certain classes of teeth, tin could be used with great advantage. As a temporary stopping, in certain cases, there was no material equal to it. But when it was associated with gold, he could not see any advantages which the union could give rise to.

The next and last paper was entitled "Mechanical Abrasion of the Anterior Teeth," by Dr. E. L. Hunter, Enfield, North Carolina, in which cases the Doctor advocated the use of a frame with the coin foil for building up the tooth.

Dr. A. P. Merrill said that if he understood the paper rightly, Dr. Hunter's method was to use gold coin with the foil. He thought in cases where the tooth was built up from the root, and where there was a sufficient hold to groove, that, by properly grooving, operations could be performed that would wear as long as so much solid gold. He had a case of the kind in wear since 1864. In another case, where there were four central incisors filled, (where most of the molar and bicuspid teeth had been extracted, while those remaining were accluded;) he had had them in wear since 1869, four gold crowns which he had built up from the margin of the gum, giving them their proper shape.

Dr. A. McCollom said he had a case in point. He had filled a central incisor, in the way Dr. Merrill referred to, eight or nine years back, and it was now doing service. He was afraid that Dr. Hunter did not use the automatic mallet, and work the gold enough. If the gold was thoroughly condensed, he would guarantee it to last as long as American coin.

Dr. J. R. Gober said that Dr. Hunter displayed in his paper very nice mechanical skill; but he thought that the Doctor's method did not pay. It complicated the operation very much, and he had yet to see a properly condensed filling wear away any faster than gold plates wear away in the mouth. He had a case of a left central incisor, the crown one half gone an approximal cavity above the free margin of the gum—pulp alive, filled four years ago with No. 4 soft foil annealed, and condensed with an eight ounce mallet in the hands of a good assistant; and that filling was there to day as good as when first put in.

After some further discussion, the meeting adjourned.

